# The Effect of COVID-19 on Mental Health and Wellbeing in a Representative Sample of Australian Adults

**DOI:** 10.3389/fpsyt.2020.579985

**Published:** 2020-10-06

**Authors:** Amy Dawel, Yiyun Shou, Michael Smithson, Nicolas Cherbuin, Michelle Banfield, Alison L. Calear, Louise M. Farrer, Darren Gray, Amelia Gulliver, Tambri Housen, Sonia M. McCallum, Alyssa R. Morse, Kristen Murray, Eryn Newman, Rachael M. Rodney Harris, Philip J. Batterham

**Affiliations:** ^1^ Research School of Psychology, The Australian National University, Canberra, ACT, Australia; ^2^ Centre for Research on Ageing, Health and Wellbeing, Research School of Population Health, The Australian National University, Canberra, ACT, Australia; ^3^ Centre for Mental Health Research, Research School of Population Health, The Australian National University, Canberra, ACT, Australia; ^4^ Department of Global Health, Research School of Population Health, The Australian National University, Canberra, ACT, Australia; ^5^ National Centre for Epidemiology and Population Health, Research School of Population Health, The Australian National University, Canberra, ACT, Australia

**Keywords:** coronavirus, COVID-19, bushfire, mental health, anxiety, depression, financial strain

## Abstract

There is minimal knowledge about the impact of large-scale epidemics on community mental health, particularly during the acute phase. This gap in knowledge means we are critically ill-equipped to support communities as they face the unprecedented COVID-19 pandemic. This study aimed to provide data urgently needed to inform government policy and resource allocation now and in other future crises. The study was the first to survey a representative sample from the Australian population at the early acute phase of the COVID-19 pandemic. Depression, anxiety, and psychological wellbeing were measured with well-validated scales (PHQ-9, GAD-7, WHO-5). Using linear regression, we tested for associations between mental health and exposure to COVID-19, impacts of COVID-19 on work and social functioning, and socio-demographic factors. Depression and anxiety symptoms were substantively elevated relative to usual population data, including for individuals with no existing mental health diagnosis. Exposure to COVID-19 had minimal association with mental health outcomes. Recent exposure to the Australian bushfires was also unrelated to depression and anxiety, although bushfire smoke exposure correlated with reduced psychological wellbeing. In contrast, pandemic-induced impairments in work and social functioning were strongly associated with elevated depression and anxiety symptoms, as well as decreased psychological wellbeing. Financial distress due to the pandemic, rather than job loss *per se*, was also a key correlate of poorer mental health. These findings suggest that minimizing disruption to work and social functioning, and increasing access to mental health services in the community, are important policy goals to minimize pandemic-related impacts on mental health and wellbeing. Innovative and creative strategies are needed to meet these community needs while continuing to enact vital public health strategies to control the spread of COVID-19.

## Introduction

The new coronavirus SARS-CoV-2 (COVID-19) pandemic is unprecedented in recent history, with global impacts including high rates of mortality and morbidity, and loss of income and sustained social isolation for billions of people. The effect this crisis will have on population mental health, both in the short- and long-term, is unknown. There is minimal evidence about the acute phase mental health impacts of large-scale epidemics across communities. Existing work has focused on those individuals most directly affected by disease (e.g., infected individuals and their families, healthcare workers (1–5) and examined mental health impacts across broader communities only after the acute phase has passed (1). In the acute phase however, fear about potential exposure to infection, loss of employment, and financial strain are also likely to increase psychological distress in the broader population (1–4). This distress may be further exacerbated in individuals who have experienced prior traumatic events (2). In the longer term, grief and trauma are likely to emerge (3) and, as financial and social impacts become entrenched, risk of depression and suicidality may increase (2, 6–8).

Reports of the mental health impacts of previous severe health epidemics have focused primarily on disease survivors [e.g., of Ebola virus disease (2) and SARS (1)]. Almost invariably, these studies show survivors experience greater psychological distress post-epidemic than others from affected communities (1, 3). Risk for psychological distress may also be greater for people employed in occupations that potentially expose them to infection (4, 5), and in those who have friends or family members who have been infected (3). However, in the acute phase of COVID-19, there are clear reasons to also expect that Government policies and physical distancing measures aimed at limiting disease spread will impact mental health in the broader community. For instance, loss of employment (6), financial strain (9), and social isolation (8, 10) are all well-documented correlates of mental health problems. In many countries, physical distancing measures have already resulted in an enormous increase in unemployment (11), likely causing significant financial strain for many.

**Figure 1:**
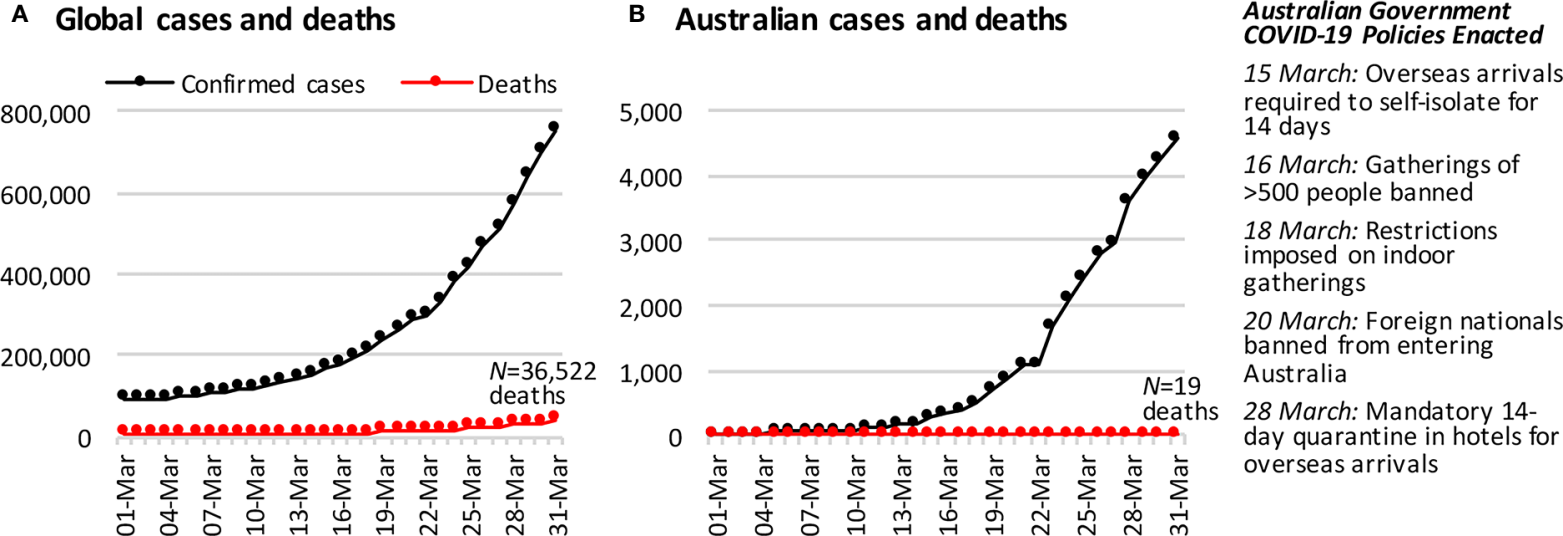
The cumulative number of COVID-19 confirmed cases and deaths **(A)** across the globe and **(B)** in Australia, in the month leading up to the first survey wave of this study. Case and death data are from https://covid19.who.int/.

Gathering early evidence of the impacts of COVID-19 is vital for informing mental health service delivery as the pandemic and its extended effects continue. The present study surveyed a representative sample of Australians from 28 to 31 March 2020, during the acute phase of the pandemic in Australia. **Figure 1** shows the number of confirmed cases in Australia had just started to escalate at this time, relative to global cases. A total of 19 deaths had been reported in Australia by the survey close, relative to over 36,500 across the globe. In the fortnight leading up to the survey, the Australian government had closed restaurants, bars, and churches, severely restricted the size of public and private gatherings, banned foreign nationals from entering Australia, and was enforcing strict quarantine measures for Australians returning from overseas.

The present study aimed to document the initial mental health scenario across the Australian community and examine its association with exposure to the broad COVID-19 environment at this critical acute phase by: (1) measuring the current prevalence of clinically significant symptoms of generalized anxiety and depression, including associations with other recent adversities; and (2) investigating the degree to which symptom severity is associated with exposure to COVID-19, and pandemic-related impacts on employment, finances, and social functioning. We also accounted for exposure to the catastrophic bushfires that occurred across Australia in November 2019–January 2020. We hypothesized that greater exposure to COVID-19, and impairment in employment, finances, and social functioning, would be associated with higher psychological distress and decreased psychological wellbeing

## Methods

### Study Design and Sample

We established a new longitudinal study—The Australian National COVID-19 Mental Health, Behavior and Risk Communication (COVID-MHBRC) Survey—to investigate the impact of the COVID-19 pandemic on a representative sample of the Australian adult population (≥18 years). Participants were required to be able to respond to an online English language survey. The study comprises seven survey waves initiated online fortnightly, *via* Qualtrics Research Services. Recruitment was conducted using quota sampling to obtain a representative sample on the basis of age group, gender, and geographical location (State/Territory). Participants gave written informed consent after receiving a complete description of the study. The study was approved by The Australian National University Human Research Ethics Committee (number 2020/152). The full study protocol is available here: https://psychology.anu.edu.au/files/COVID_MHBRCS_protocol.pdf.

We report data (N = 1,296) from the first assessment (Wave 1, 28–31 March 2020). The sample size requirement estimate was based on planned power analyses for finding an effect of *f* ^2^ = 0.1 in linear and logistic regression models, setting 1 - *β* = .95 and *α* = .05, and taking into account variations in the prevalence of binary outcomes and attrition over the stages of the longitudinal survey, and an allowance for 10% unusable data. Our sample of *N* = 1,296 was only 2% less than our target sample of *N* = 1,320 (see **Supplement S1** for additional details). Only 2–3% of the data were unusable for the present analyses.

**Table 1** reports Wave 1 sample distributions by gender, age, and location. These distributions aligned well with population data from the Australian Bureau of Statistics (12), demonstrating that a representative sample of the Australian community was achieved.

**Table 1:** Sample demographics and comparison with population data from the 2016 Australian Census (12).

		Sample *n*	%	Population %
Gender				
	Male	645	49.8	49.3
	Female	649	50.2	50.7
	Missing	2		
Age				
	18–24	163	12.6	10.3
	25–34	244	18.8	18.8
	35–44	231	17.8	17.6
	45–54	223	17.2	17.3
	55–64	195	15.0	15.4
	65+	240	18.5	20.5
State/Territory				
	Australian Capital Territory	37	2.9	1.6
	New South Wales	409	31.6	32.2
	Northern Territory	12	0.9	1.0
	Queensland	249	19.2	20.3
	South Australia	96	7.4	7.3
	Tasmania	36	2.8	2.3
	Victoria	313	24.2	24.9
	Western Australia	144	11.1	10.4

### Survey Measures

Symptoms of depression and anxiety over the last 2 weeks were assessed by the Patient Health Questionnaire-9 (PHQ-9) (13) and Generalized Anxiety Disorder-7 (GAD-7) (13) respectively. These measures align closely with diagnostic criteria for major depressive disorder and generalized anxiety disorder respectively (14). General psychological wellbeing over the last 2 weeks was measured using the World Health Organization Wellbeing Index (WHO-5) (15).

COVID-19 exposure was computed as the sum of self-reports of possible or actual exposures to the virus, of the related population health response, or of close social impact including: having been diagnosed with the virus, awaiting results from a test, having tested negative to the test, being in direct contact with a carrier of the virus, having had to isolate in the past, having chosen to isolate in the past, being currently forced to isolate, currently choosing to isolate, having a family member diagnosed with the virus, having a family member in isolation, knowing someone who was diagnosed, knowing someone in isolation, or being asked to work from home because of the virus.

Our measures of the work and social impacts of COVID-19 were whether someone had lost their job due to COVID-19 (yes/no); was working from home due to COVID-19 (yes/no); was experiencing financial distress due to COVID-19 (six-point Likert-type rating, from Not at all to Extremely); and the overall extent to which their work and social activities were impaired by COVID-19, measured using the Work and Social Adjustment Scale (WSAS) (16). For the WSAS, participants rated the level of impairment COVID-19 had caused (eight-point Likert-type rating, from Not at all impaired to Very severely impaired) for five work and social domains (ability to work, home management, social leisure activities, private leisure activities, and ability to form and maintain close relationships).

We also measured other background factors that could be associated with mental health: age (in years); gender (male/female/other); years of education; partner status (yes/no); living alone (yes/no); living with dependent children (yes/no); existing health, neurological, or psychological conditions, diagnosed by an appropriate clinician (yes/no); recent exposure to bushfire smoke (yes/no) or fire (yes/no); and impact of other recent adverse life events (five-point Likert-type rating, from Not at all to Extremely). Regarding the bushfire exposure variables, our reason for separating out smoke from fire is that many Australians who were exposed to smoke lived far away from the actual fires and their home/region was never under direct threat. The major impact for smoke-but-not-fire affected individuals was poor air quality, which prohibited people from spending time outside for several weeks over the Summer.

### Statistical Analysis

Statistical analyses were conducted in R version 3.6.3 under RStudio version 1.1.456 (17). Multiple linear regression was the primary technique employed to assess correlates of poor mental health. Models were checked and showed an absence of multicollinearity, outliers, and non-normality in the residuals. However, as is typical in non-clinical samples, the PHQ-9 and GAD-7 variables had high frequencies at their lowest possible values, resulting in incorrigible skew. Therefore, compound Poisson-gamma (Tweedie distribution) generalized linear models (18) were estimated as a check on the linear models (**Supplement S2**). Their results were consistent with the linear models. Likewise, the models included categorical predictors with small subsample sizes, so cross-validation was conducted to ensure that the models were stable (**Supplement S3**). Overall, <1% of data were missing. Models reported in the main text dealt with these cases using listwise deletion. We also multiply imputed the missing values and reran the models, which produced the same pattern of findings (**Supplement S5**).

## Results

**Table 2** presents our sample characteristics. Overall, 20.3 and 16.4 of our sample scored above the clinical cut-offs on our depression (PHQ-9) and anxiety (GAD-7) measures respectively. **Table 3** shows these rates are notably elevated compared to other community-based samples. Even among individuals without a current diagnosis, the rates remained elevated well above levels seen in other representative community-based samples.

**Table 2:** Description of sample characteristics, including comparison of men and women.

Sociodemographic and background factors		Whole sample (n = 1,296)		Men (n = 645)		Women (n = 649)		*t* or χ2	*df*	*p*
	Age, *M* years (SD)	46.0	(17.3)	49.5	(18.2)	42.7	(15.6)	7.17	1293	<.001***
	Education, *M* years (SD)	13.8	(2.6)	13.6	(2.7)	13.9	(2.5)	−1.68	1282	.093
	Has partner, n (%)	853	(66.2%)	421	(65.7%)	432	(67.0%)	0.19	1	.665
	Lives alone, n (%)	157	(12.1%)	82	(12.7%)	75	(11.6%)	0.30	1	.581
	Child at home, n (%)	406	(31.3%)	196	(30.4%)	209	(32.2%)	0.42	1	.519
	Any chronic disease, n (%)	503	(38.8%)	286	(44.3%)	217	(33.4%)	15.73	1	<.001***
	Any neurological disorder, n (%)	159	(12.3%)	86	(13.3%)	73	(11.3%)	1.12	1	.290
	Any mental health disorder, n (%)	310	(23.9%)	144	(22.3%)	165	(25.4%)	1.54	1	.214
**Recent adversity**										
	Bushfire exposure—smoke, n (%)	607	(46.8%)	290	(45.0%)	316	(48.7%)	1.66	1	.198
	Bushfire exposure—fire, n (%)	111	(8.6%)	66	(10.2%)	45	(6.9%)	4.08	1	.043*
	Other adverse life event, n (%)	282	(21.8%)	156	(24.2%)	126	(19.4%)	4.05	1	.044*
**COVID-19 exposure**										
	COVID-19 exposure, *M* (SD)	0.78	(0.88)	0.71	(0.82)	0.85	(0.9)	−2.75	1293	.006**
**Work and social impacts of COVID-19**										
	Working from home, n (%)	173	(13.4%)	78	(12.1%)	95	(14.6%)	1.60	1	.206
	Lost job, n (%)	117	(9.0%)	50	(7.8%)	67	(10.3%)	2.30	1	.130
	Financial distress, n (%)	652	(50.3%)	314	(48.7%)	338	(52.1%)	1.36	1	.243
	WSAS, n (SD)	20.5	(9.3)	20.3	(9.8)	20.8	(8.8)	−1.11	1293	.267
**Mental health measures**										
	PHQ-9, score (SD)	5.4	(5.9)	4.7	(5.7)	6.0	(6.0)	−3.93	1290	<.001***
	GAD-7, score (SD)	4.4	(5.2)	3.7	(4.9)	5.1	(5.4)	−5.07	1288	<.001***
** **	WHO-5, score (SD)	11.9	(5.9)	12.9	(6.0)	10.9	(5.7)	6.16	1289	<.001***

*p < .05; **p < .001; ***p < .001.

Bold indicates tests significant at p < .05.

**Table 3:** Prevalence of depression and generalized anxiety based on self-reported current mental health diagnosis.

	**Existing current diagnosis** **(*****n*** **=** **310)**		**No diagnosis** **(*****n*** **=** **985)**		**Total sample** **(*****n*** **=** **1,295)**		**Comparison to other population sample studies**
**Major Depressive Disorder (PHQ-9≥10)**	145	(46·8%)	118	(12.0%)	263	(20.3%)	5·6% (19), 6·7% (20)
**Generalized Anxiety Disorder (GAD-7≥10)**	113	(36·5%)	99	(10.1%)	212	(16.4%)	5·1% (21)

*Comparisons samples are general population samples from the USA (19) and Germany (20, 21). To the best of our knowledge, there are no published pre-pandemic norms from the Australian national population for these measures*.

Investigation of the relationships between our predictor measures and three mental health outcome measures used a Bonferroni adjusted significance threshold of 0.17 to control for the three sets of comparisons, i.e., *α* = .05/3 = .017. Note, all three measures showed good reliability (see **Supplement S6**).

Our initial univariate tests revealed that higher levels of depression and anxiety symptoms, and lower psychological wellbeing (WHO-5), were all associated with job loss and financial distress, and overall work and social impairment due to COVID-19, as measured by the WSAS. Being required to work from home was not associated with any mental health effects at this acute stage of the pandemic, all ps > 0.27 (see **Supplement S6** for all univariate results).

The linear regression models, presented in **Table 4**, established that the effects of financial distress and overall work and social impairment were independent, and not better accounted for by demographic or other background factors. Job loss however did not have a significant independent association with mental health after accounting for financial distress and other covariates, all ps > 0.25.

**Table 4:** Linear regression models for each mental health outcome.

				PHQ-9 (*n* = 1,273, *df* = 16, 1256)			GAD-7 (*n* = 1,270, *df* = 16, 1253)			WHO-5 (*n* = 1,271, *df* = 16, 1254)		
				estimate		*p*	estimate		*p*	estimate		*p*
**Constant**				3.73		<.001***	2.36		.012*	12.41		<.001***
**Sociodemographic and background factors**												
		Age		−0.05		<.001***	−0.04		<.001***	0.03		.002**
		Gender		0.84		.003**	1.02		<.001***	−1.76		<.001***
		Education		−0.10		.055	−0.04		.361	0.14		.022
		Has partner		−0.47		.150	0.14		.627	0.60		.106
		Lives alone		0.23		.628	−0.14		.739	−0.26		.627
		Child at home		−0.28		.359	−0.03		.928	0.53		.126
		Any chronic disease		0.64		.052	0.54		.072	−0.83		.026
		Any neurological disorder		1.29		.006**	0.42		.320	−0.49		.352
		Any current MH disorder		4.65		<.001***	3.92		<.001***	−3.07		<.001***
**Recent adversity**												
	Bushfire exposure—smoke			0.26		.336	0.15		.534	−0.96		.002**
	Bushfire exposure—fire			−0.40		.406	−0.48		.282	0.72		.188
	Other adverse life event			1.80		<.001***	1.31		<.001***	−0.32		.411
**COVID-19 exposure**												
	COVID-19 exposure			0.24		.129	0.18		.210	0.39		.028
**Work and social impacts of COVID-19**												
	Lost job		0.43			.383	0.51		.255	−0.24		.660
	Financial distress		2.32			<.001***	2.10		<.001***	−2.38		<.001***
	WSAS		0.09			<.001***	0.06		<.001***	−0.06		.005**
			***R^2^***		**Adjusted *R^2^***	***F***	***R^2^***	**Adjusted *R^2^***	***F***	***R^2^***	**Adjusted *R^2^***	***F***
**Model**			.369		.361	45.91***	.322	.314	38.48***	.208	.198	20.53***

*p < .017. **p < .001. ***p < .001. Bold indicates tests significant at p < .017.

In contrast, the regression analyses found no significant unique association between exposure to COVID-19 and depression or anxiety symptoms, or wellbeing.

Depression and anxiety symptoms were also elevated in people who had experienced other recent adversities, although this did not include direct exposure to the recent catastrophic Australian bushfires. Exposure to bushfire smoke was however associated with decreased wellbeing.

Finally, within these regression models, we also found that younger age, identifying as female, and having at least one current mental health disorder were each independently associated with higher levels of depression and anxiety, and decreased wellbeing.

## Discussion

We found the social, work, and financial disruptions induced by the acute phase of the COVID-19 pandemic were associated with considerable impairments in community mental health in Australian adults. In contrast, exposure to COVID-19 was not found to predict mental health in this cohort. A key strength of this study was the testing of a representative community sample early in the pandemic, providing rapid evidence of population mental health status. The results highlight that epidemics may cause serious problems for community mental health in the acute phase of disease.

Indeed, our results suggest that, at a population level, changes to social and work functioning due to COVID-19 were more strongly associated with decrements in mental health than amount of disease contact. This finding is consistent with a recent UK-based finding that their citizens were more concerned about how societal changes will impact their psychological and financial wellbeing, than becoming unwell with the virus (7). This finding is also consistent with emergent work indicating that loneliness is playing a central role in the observed mental health impacts of the COVID-19 pandemic (22–24). Altogether then, it is evident that the necessary public health arrangements surrounding the pandemic are having serious implications for community mental health, *via* their disruption to social and work functioning.

However, this does not mean the mental health costs of pandemic-related social changes will inevitably be greater than those caused by exposure to disease. In Australia, mortality rates were very low at the time of this study, and the health system had capacity to meet demand. The relatively low case rates were also reflected in our sample; although the majority of the sample had some exposure, such as needing to self-isolate, only 36 participants reported direct exposure to the virus (self or close contact diagnosed). The short-term mental health impacts of disease contact may be considerably greater in communities that have high mortality rates, and health systems over-burdened by disease. In the longer-term, disease contact may also lead to elevated levels of trauma and grief for affected individuals (3).

The elevated levels of psychological distress observed in this study indicate mental health services are likely to experience increased demand during pandemics. Following recommended physical distancing guidelines, these will need to be delivered flexibly, leveraging resources for telehealth and internet-based Cognitive Behavior Therapy (CBT) programs, which have been shown to be effective in preventing and treating common mental disorders (7, 25, 26). There may also be an increased role for community cohesion strategies (27) and peer support (28), for instance, drawing on the experience and knowledge of people already living with mental health issues to support those experiencing these issues for the first time.

The findings also provide clear evidence that minimizing social and financial disruption during the COVID-19 pandemic should be a central goal of public health policy. A key challenge is how to best achieve this goal without compromising public safety by, for instance, relaxing physical distancing restrictions too early. Our results suggest policy approaches that target financial support to those experiencing financial strain may be useful, rather than on the basis of lost employment alone. We also found that well-established risk factors for poorer mental health—younger age, identifying as female, and having a pre-existing mental health condition—continue to be associated with increased risk within the pandemic context. Governments should consider additional measures to monitor and support these at-risk groups. Psychosocial interventions to support multiple aspects of wellbeing, including minimizing financial debt, may have positive impacts on depression and anxiety in the community (29). Clinicians should also remain vigilant for potential added social and financial impacts that existing clients in primary care and psychological settings may be experiencing.

A possible limitation of the present study is the use of self-report scales that may not characterize mental health status with the accuracy of structured clinical interviews, although both the PHQ-9 and GAD-7 have previously demonstrated strong alignment with clinical diagnosis in population samples (14), and the WHO-5 is also well-validated (15). Another potential issue is the influence of selection bias on the prevalence of mental health problems seen in this sample, however, the likelihood of this is low. We were careful to ensure the recruitment advertisement did not mention the topic or nature of our survey (e.g., no mention of mental health or COVID-19 at all), and the service we used also recruits participants for non-psychological research (i.e., market research panel). Most importantly, we did obtain a sample that was representative of the Australian population by age, gender, and location. It is however important to note that online survey methods may bias samples towards people who have good internet literacy and access (30). This type of bias may have a disproportionate impact on subsections of the population, such as older adults.

Finally, this initial report of our work is cross-sectional. The observed associations may not reflect causal effects, and the nature of any causal relationships may be more complicated than our interpretation suggests (e.g., possible bi-directional effects between psychological distress and social/occupational functioning). We intend to balance the necessity of providing our first wave findings in a timely fashion, to rapidly inform ongoing global responses to the pandemic, by reporting longitudinal outcomes as they become available in the coming months. Examination of population subgroups within our sample may also be possible in longitudinal analyses, although additional targeted studies may be required to provide greater insight into how specific vulnerable groups are affected. These findings should also be considered in combination with other studies that survey the mental health impacts of COVID-19 in communities that have adopted different approaches to managing the pandemic and/or have differing social structures (e.g., low GDP) to Australia.

In conclusion, the current study provides a snapshot of the acute phase impact of COVID-19 on the mental health of the Australian adult community. The findings are concerning, suggesting markedly elevated rates of depression and anxiety, even among individuals with no current diagnosis. This worsening of mental health may also have been exacerbated by the recent severe bushfire season Australians had experienced in the months leading up to the pandemic, although bushfire exposure was controlled for in our analyses. Overall, the findings suggest that interventions to counteract the social, financial and role disruptions induced by COVID-19, particularly among people with existing health conditions, are likely to have the greatest impact on community mental health and wellbeing.

## Data Availability Statement

The raw data supporting the conclusions of this article will be made available by the authors, without undue reservation.

## Ethics Statement

The studies involving human participants were reviewed and approved by the Australian National University Human Research Ethics Committee. The patients/participants provided their written informed consent to participate in this study.

## Author Contributions

All authors contributed to the design and conceptualization of the study, which was coordinated by AD. AD, PJB, and LMF contributed to the literature review. AD, PJB, YS, MS, and NC contributed to the data analyses and formulation of the manuscript, with input from all other authors. AD, PJB, NC, and MS drafted the manuscript and all authors critically revised the manuscript. All authors contributed to the article and approved the submitted version.

## Funding

This study was funded by the ANU College of Health and Medicine, ANU Research School of Psychology, and ANU Research School of Population Health. PJB is supported by National Health and Medical Research Council (NHMRC) Fellowship 1158707. ALC is supported by NHMRC Fellowships 1122544 and 1173146. LMF is supported by Australian Research Council Discovery Early Career Researcher Award (ARC DECRA) DE190101382. YS is supported by ARC DECRA DE180100015. AG and ARM are supported by funding provided by the ACT Health Directorate for ACACIA: The ACT Consumer and Carer Mental Health Research Unit.

## Conflict of Interest

The authors declare that the research was conducted in the absence of any commercial or financial relationships that could be construed as a potential conflict of interest.
