# Corrigendum: The Effect of COVID-19 on Mental Health and Wellbeing in a Representative Sample of Australian Adults

**DOI:** 10.3389/fpsyt.2020.619331

**Published:** 2021-01-21

**Authors:** Amy Dawel, Yiyun Shou, Michael Smithson, Nicolas Cherbuin, Michelle Banfield, Alison L. Calear, Louise M. Farrer, Darren Gray, Amelia Gulliver, Tambri Housen, Sonia M. McCallum, Alyssa R. Morse, Kristen Murray, Eryn Newman, Rachael M. Rodney Harris, Philip J. Batterham

**Affiliations:** ^1^Research School of Psychology, The Australian National University, Canberra, ACT, Australia; ^2^Centre for Research on Ageing, Health and Wellbeing, Research School of Population Health, The Australian National University, Canberra, ACT, Australia; ^3^Centre for Mental Health Research, Research School of Population Health, The Australian National University, Canberra, ACT, Australia; ^4^Department of Global Health, Research School of Population Health, The Australian National University, Canberra, ACT, Australia; ^5^National Centre for Epidemiology and Population Health, Research School of Population Health, The Australian National University, Canberra, ACT, Australia

**Keywords:** coronavirus, COVID-19, bushfire, mental health, anxiety, depression, financial strain

In the original article, there was an error in Table 3 as published. The prevalence of depression and generalized anxiety in our sample appeared in the wrong rows. The main text of the original article did however report the prevalence rates correctly, i.e., the statement “Overall, 20.3% and 16.4% of our sample scored above the clinical cut-offs on our depression (PHQ-9) and anxiety (GAD-7) measures respectively.” (p. 4 of original article) is correct. The corrected Table 3 appears below.

**Table 3:** Prevalence of depression and generalized anxiety based on self-reported current mental health diagnosis.

	**Existing current diagnosis** **(*****n*** **=** **310)**		**No diagnosis** **(*****n*** **=** **985)**		**Total sample** **(*****n*** **=** **1,295)**		**Comparison to other population sample studies**
Major Depressive Disorder (PHQ-9≥10)	145	(46·8%)	118	(12.0%)	263	(20.3%)	5·6% (19), 6·7% (20)
Generalized Anxiety Disorder (GAD-7≥10)	113	(36·5%)	99	(10.1%)	212	(16.4%)	5·1% (21)

*Comparisons samples are general population samples from the USA (19) and Germany (20, 21). To the best of our knowledge, there are no published pre-pandemic norms from the Australian national population for these measures*.

The authors apologize for this error and state that this does not change the scientific conclusions of the article in any way. The original article has been updated.

