# Supplementary material for: The Effect of COVID-19 on Mental Health and Wellbeing in a Representative Sample of Australian Adults
Source: Front Psychiatry. 2020 Oct 6;11:579985. doi: 10.3389/fpsyt.2020.579985 (PMC7573356; doi:10.3389/fpsyt.2020.579985)
Supplement: Supplementary file 1 [file DataSheet_1.pdf]

Supplementary Material

The effect of COVID-19 on mental health and wellbeing in a representative sample of Australian adults

Dr Amy Dawel*[1], Dr Yiyun Shou[1], Prof Michael Smithson[1], Prof Nicolas Cherbuin[2], Dr Michelle Banfield[3], Assoc Prof Alison L. Calear[3], Dr Louise M. Farrer[3], Prof Darren Gray[5], Dr Amelia Gulliver[3], Dr Tambri Housen[4], Dr Sonia M. McCallum[3], Dr Alyssa R. Morse[3], Dr Dr Kristen Murray[1], Eryn Newman[1], Dr Rachael M. Rodney Harris[4], Prof Philip J. Batterham[3]

[1]Research School of Psychology, The Australian National University, Canberra, ACT, Australia.

[2]Centre for Research on Ageing, Health and Wellbeing, Research School of Population Health, The Australian National University, Canberra, ACT, Australia.

[3]Centre for Mental Health Research, Research School of Population Health, The Australian National University, Canberra, ACT, Australia.

[4]National Centre for Epidemiology and Population Health, Research School of Population Health, The Australian National University, Canberra, ACT, Australia.

[5]Department of Global Health, Research School of Population Health, The Australian National University, Canberra, ACT, Australia.

*** Correspondence:**
Dr Amy Dawel
amy.dawel@anu.edu.au

Supplement S1 — Power Analysis and Sample Size Estimates

The approach adopted here for power analysis and sample size requirements estimation takes into account the primary multivariate techniques employed and attrition over 6 stages of the longitudinal survey.

Multiple Linear Regression

The sample size estimates in Figure 1 are for fixed-score regression models assuming that there are 10 predictors in the model, $\alpha = .05$, and $1 - \beta = .95$. The effect-size being used is

$$f^2 = R^2/\left(1 - R^2\right).$$

In Figure 1, f^2 varies from .05 to .15, which is the same as having R^2 vary from .048 to .130. Any model with fewer predictors and/or larger effect-sizes will require smaller samples than these to detect with power of .95. We assume that we will be evaluating regression models with no more than 10 predictors, so the power estimates in Figure S1a are based on a regression model with 10 predictors.

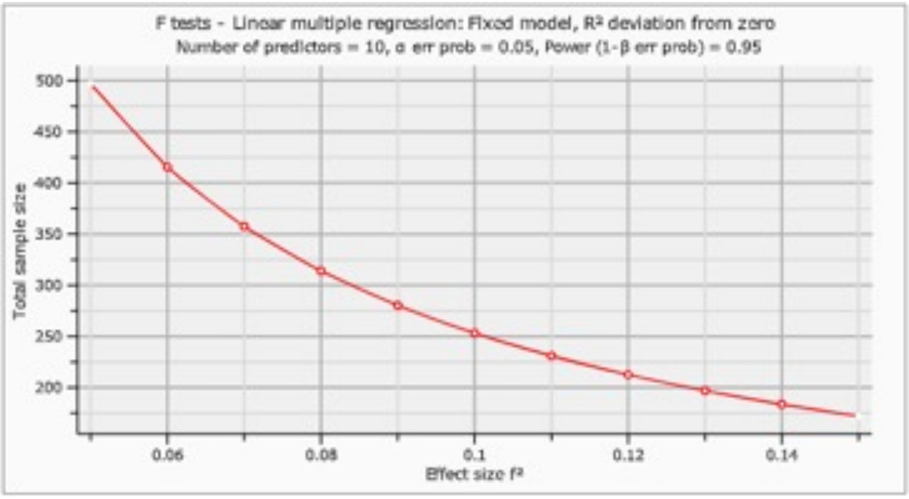

Figure S1a. Multiple Regression Sample Size Requirements

Logistic Regression

Logistic regression also will be employed, so we investigate sample size requirements for it as well. Power analysis for binary logistic regression is not as straightforward as it is for linear regression. In addition to the specified α, $1 - \beta$, and effect-size (odds-ratio), it also depends on the probability-split for the dependent variable. Figure S1b displays sample-size calculations for two scenarios, in both of which $\alpha = .05$, $1 - \beta = .95$, and the odds-ratio to be detected ranges from 1.25 to 1.75. In the top graph, the dependent variable split is 0.25/0.75, whereas in the middle graph it is 0.5/0.5. As would be expected, the sample size requirements are greater for the uneven split.

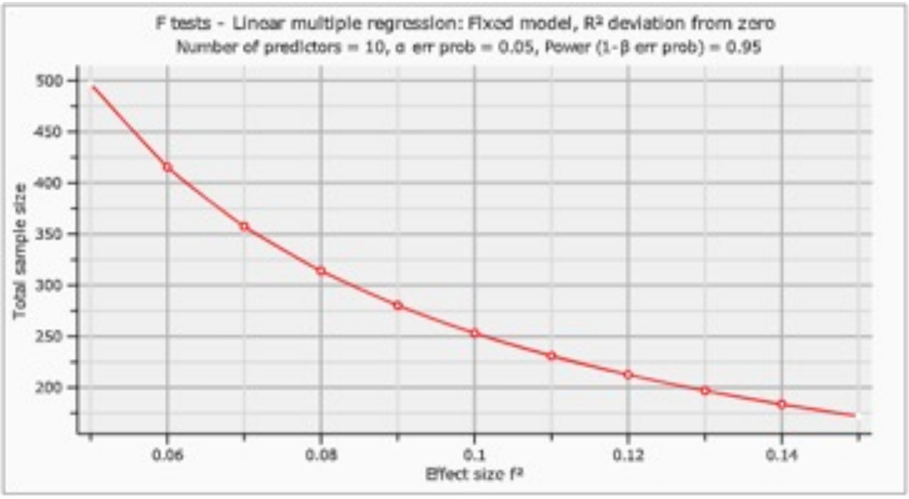

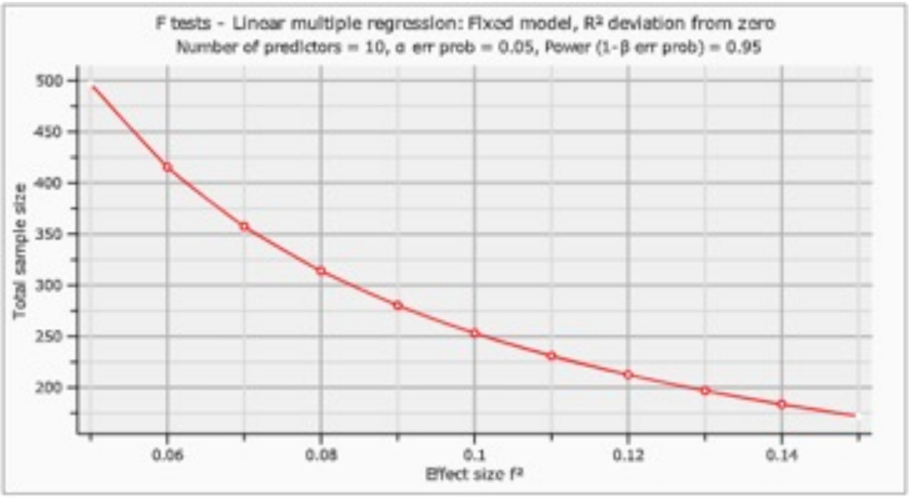

Figure S1b. Logistic Regression Sample Size Requirements

Sample Size Requirement Estimation

The final specification needed to estimate sample size is the minimum effect-size of interest. In the regression models, we take $f^2 = 0.1$ as our benchmark, and the graph in Figure S1a indicates a sample size of 250 will suffice. For the logistic regressions, a sample size of 250 suffices for detecting odds-ratios as low as about 1.55 when the dependent variable split is 0.5/0.5 and 1.65 when it is 0.25/0.75.

Assuming a sample size of 250 at the 7th (final) stage of the longitudinal survey, we may estimate the required initial sample size, given an attrition rate and allowing for a safety-margin due to unusable data. Based on extensive past experience with the crowd-sourcing platform we employ, we anticipate an overall loss of 10% of data due to unusable responses. Based on experience with other similar longitudinal survey projects, we anticipate an attrition rate of 23% at each stage. The estimated initial sample size therefore is

$$N = 1.1\left(250/.77^6\right) = 1319.4$$

or approximately 1320.

Supplement S2 — List of non-standardised measures

- What is your age in years?

- What is your gender?
    - *Male/ Female/ Other/Prefer not to say*

- Are you currently in a relationship with someone?
    - Yes, living with the person you are married to
    - Yes, living with a partner (but not married to them)
    - Yes, in a relationship with someone but not living with them
    - No, not in a relationship with anyone
    - Prefer not to say

- Do any of the following currently live in your household?
    - Spouse/partner
    - Any of your children
    - A parent or parent-in-law
    - A grandparent
    - A brother or sister
    - A son-in-law or daughter-in-law
    - A grandchild
    - Other relatives
    - Someone who is not a relative
    - Pets
    - Other

- How many dependent children do you have currently living in your household?
- What is the highest qualification you have completed?
    - School certificate (or equivalent)
    - Higher school certificate (or equivalent)
    - Trade certificate/apprenticeship
    - Technicians certificate/advanced certificate
    - Certificate other than above
    - Associate diploma
    - Undergraduate diploma
    - Bachelor degree
    - Post graduate diploma/certificate
    - Higher degree
    - Prefer not to say

Note, years of education was estimated by adapting responses to highest level of education.

- Are you currently, or have you ever been, diagnosed by an appropriate clinician with any of the following medical conditions?
  - Hypertension; Heart disease; Type 1 diabetes; Type 2 diabetes; Asthma; Chronic obstructive pulmonary disease; Kidney disease; Epilepsy; Stroke; Multiple sclerosis; Parkinson's disease; Dementia; Liver disease; Gastrointestinal condition; Joint/muscle condition; Chronic pain; Chronic fatigue syndrome; Cancer; Severe/life-threatening allergy

- Are you currently, or have you ever been, diagnosed by an appropriate clinician with any of the following psychological conditions?
  - Anxiety; Depression; Bipolar disorder; Schizophrenia; Post-traumatic stress disorder; Autism spectrum disorder; Alcohol or substance disorder; Eating disorder; Other (specify)

- To what extent were you affected by bushfires in 2019-2020?
  - Not at all
  - Some smoke
  - Heavy smoke
  - Fire within 5km of residence
  - I was evacuated due to bushfire
  - I had direct contact with bushfire
  - Fire damaged residence
  - Fire destroyed residence
  - Family member or close friend lost property or was injured
  - I was injured by fire
  - Other (please specify)

- Excluding events related to COVID-19 and the bushfires, to what extent have you been affected by other adverse events in 2020?
  - *Not at all/A little/Somewhat/A lot/Extremely*

- Over the last 2 weeks, have there been any changes to your employment situation as a result of COVID-19? (choose all that apply)
  - I lost my job
  - I was asked to work from home
  - I was forced to work from home

- Over the last 2 weeks, to what extent have you experienced financial distress related to COVID-19?
  - *Not at all/A little/Somewhat/Quite a lot/Considerably/Extremely*

- To what extent have you been affected by COVID-19? (choose all you have experienced to date)
    - I have been diagnosed positive for COVID-19 by a laboratory test
    - I have been tested for COVID-19–awaiting result
    - I have been tested for COVID-19–negative result
    - I was directed by the health department to self-isolate–current
    - I was directed by the health department to self-isolate–past
    - I have voluntarily self-isolated-current
    - I have voluntarily self-isolated–past
    - I have been a contact of someone who has been diagnosed positive by a laboratory test
    - I have a family member who is currently or has been required to self-isolate
    - I have a family member who has been diagnosed positive by a laboratory test
    - I know someone who has been required to self-isolate
    - I know someone who has been diagnosed positive by a laboratory test (but have had no contact while infectious)
    - Other (please specify)
    - None of the above

Supplement S3 — Compound Poisson-Gamma Models

As is often the case for non-clinical samples, the PHQ-9 and GAD-7 variables had large spikes at their lowest possible values, resulting in incorrigible skew. Figure S3a illustrates this with male and female sample histograms. Because they strongly violate the assumption of normality, it is possible that normal-theory linear regression models may be misspecified. To check for this possibility, we treated the boundary scores as true scores rather than censored scores. Therefore, instead of estimating Tobit models (which would suit censored-score dependent variables), we estimated compound Poisson-gamma (Tweedie distribution) generalized linear models (see Smithson & Shou 2019, pp. 30-34). Tweedie distributions are a broad class of distributions and they include a distribution with mass at 0 and a gamma density over the positive half of the real line. The cplm package in R (Zhang, 2013) was used to estimate the models described here.

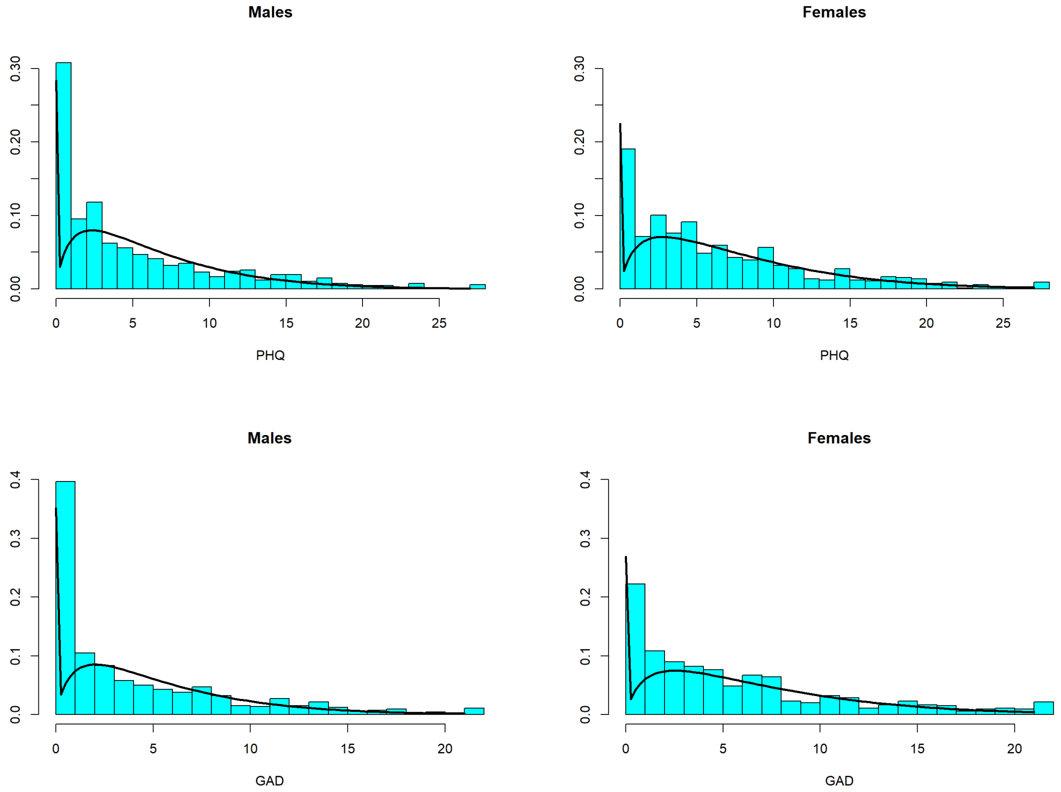

Figure S3a. Male and Female PHQ-9 and GAD-7 Histograms with Fitted Tweedie Distributions

The Tweedie GLMs were fitted to both the non-imputed and imputed versions of the data. Table S3a displays the summary statistics for the non-imputed data in the models for PHQ, GAD, and WHO-5, and Table S2b contains the output for the imputed data mode.

Table S3a. Tweedie GLMs for Non-Imputed Data

	**PHQ-9**	**N =**	**1286**		**GAD-7**	**N =**	**1286**		**WHO-5**	**N =**	**1284**	
Parameter	***B***	***SE***	***t***	***p***	***B***	***SE***	***t***	***p***	***B***	***SE***	***t***	***p***
Intercept	1.601	.271	5.908	<.001	1.178	.308	3.832	<.001	2.391	.129	18.539	<.001
Age	-.012	.002	-6.146	**<.001**	-.013	.002	-5.534	**<.001**	.003	.001	2.806	**.005**
Any current chronic health condition	.098	.066	1.491	.136	.081	.074	1.100	.272	-.066	.031	-2.115	.035
Any current mental health diagnosis	.674	.062	10.947	**<.001**	.710	.069	10.226	**<.001**	-.284	.036	-7.955	**<.001**
Any current neurological condition	.219	.082	2.658	**.008**	.122	.095	1.286	.199	-.066	.045	-1.449	.148
Bushfire exposure—fire	-.017	.091	-.183	.855	-.056	.104	-.537	.592	.046	.048	.964	.335
Bushfire exposure—smoke	.069	.055	1.249	.212	.053	.062	.856	.392	-.084	.026	-3.173	**.002**
COVID-19 exposure	.028	.032	.876	.381	.011	.036	.303	.762	.040	.016	2.527	**.012**
Education	-.030	.016	-1.877	.061	-.021	.018	-1.149	.251	.017	.008	2.322	.020
Financial distress due to COVID-19	.459	.062	7.433	**<.001**	.522	.069	7.515	**<.001**	-.182	.033	-5.600	**<.001**
Gender	.204	.056	3.629	**<.001**	.276	.064	4.345	**<.001**	-.138	.027	-5.141	**<.001**
Has partner	-.092	.067	-1.389	.165	.018	.076	.231	.817	.070	.035	1.987	.047
Lives with dependent children	-.005	.062	-.083	.934	.044	.069	.644	.519	.044	.030	1.453	.147
Lives alone	.087	.085	1.029	.304	.008	.099	.082	.935	.028	.042	.667	.505
Lost job due to COVID-19	-.011	.087	-.128	.898	.018	.098	.189	.850	-.040	.051	-.772	.440
Other adverse life event	.296	.063	4.709	**<.001**	.278	.071	3.905	**<.001**	-.028	.035	-.800	.424
Working from home due COVID-19	.006	.088	.070	.944	.116	.096	1.206	.228	-.043	.041	-1.032	.302
WSAS	.016	.003	4.858	**<.001**	.015	.004	4.013	**<.001**	-.006	.002	-3.826	**<.001**

Note. Bolded responses are significant at $p<.017$.

Table S3b. Tweedie GLMs for Imputed Data

	**PHQ-9**	**N =**	**1296**		**GAD-7**	**N =**	**1296**		**WHO5**	**N =**	**1296**	
Parameter	***B***	***SE***	***t***	***p***	***B***	***SE***	***t***	***p***	***B***	***SE***	***t***	***p***
Intercept	1.302	.211	6.166	**<.001**	.855	.238	3.593	**<.001**	2.515	.098	25.535	**<.001**
Age	-.011	.002	-5.463	**<.001**	-.011	.002	-5.036	**<.001**	.003	.001	2.844	**.005**
Any current chronic health condition	.099	.069	1.438	.151	.097	.077	1.262	.207	-.068	.032	-2.148	.032
Any current mental health diagnosis	.721	.061	11.823	**<.001**	.754	.068	11.076	**<.001**	-.290	.035	-8.281	**<.001**
Any current neurological condition	.184	.086	2.126	.034	.064	.099	.643	.520	-.054	.047	-1.160	.246
Bushfire exposure—fire	-.055	.091	-.607	.544	-.088	.104	-.847	.397	.066	.047	1.389	.165
Bushfire exposure—smoke	.043	.055	.787	.431	.026	.061	.429	.668	-.081	.026	-3.130	**.002**
Child at home	-.029	.062	-.463	.643	.027	.068	.398	.691	.046	.030	1.538	.124
COVID-19 exposure	.035	.031	1.138	.256	.027	.034	.794	.427	.033	.015	2.178	.030
Education	-.020	.011	-1.773	.077	-.008	.012	-.672	.502	.012	.005	2.323	.020
Financial distress due to COVID-19	.521	.066	7.894	**<.001**	.571	.074	7.755	**<.001**	-.197	.030	-6.546	**<.001**
Gender	.202	.056	3.583	**<.001**	.285	.063	4.522	**<.001**	-.143	.026	-5.387	**<.001**
Has partner	-.094	.063	-1.487	.137	.022	.071	.304	.761	.050	.032	1.568	.117
Lives alone	.091	.094	.966	.334	-.019	.111	-.173	.863	-.021	.045	-.456	.649
Lost job due to COVID-19	.001	.086	.016	.987	.015	.096	.154	.878	-.028	.051	-.554	.580
Other adverse life event	.294	.063	4.675	**<.001**	.272	.071	3.853	**<.001**	-.020	.034	-.569	.569
WSAS	.014	.003	4.268	**<.001**	.013	.004	3.567	**<.001**	-.005	.002	-3.126	**.002**

Note. Bolded responses are significant at $p<.017$.

The coefficients and their significance-levels show no important inconsistencies between the two types of models, although the Tweedie models are more conservative about a few "marginally significant" effects in the linear models. The fitted values of the two types of models also are strongly related, as the scatterplots show in Figure S3b. For the PHQ and GAD models, the main discrepancies are the out-of-range negative predictions by the linear regression models (28 cases for PHQ and 37 cases for GAD for the non-imputed data, and 37 cases for PHQ and 41 cases for GAD for the imputed data) and the greater ranges of fitted values for the Tweedie models. The WHO-5 models have the greatest similarity because the WHO-5 scale does not have zero-inflation.

Non-Imputed Data

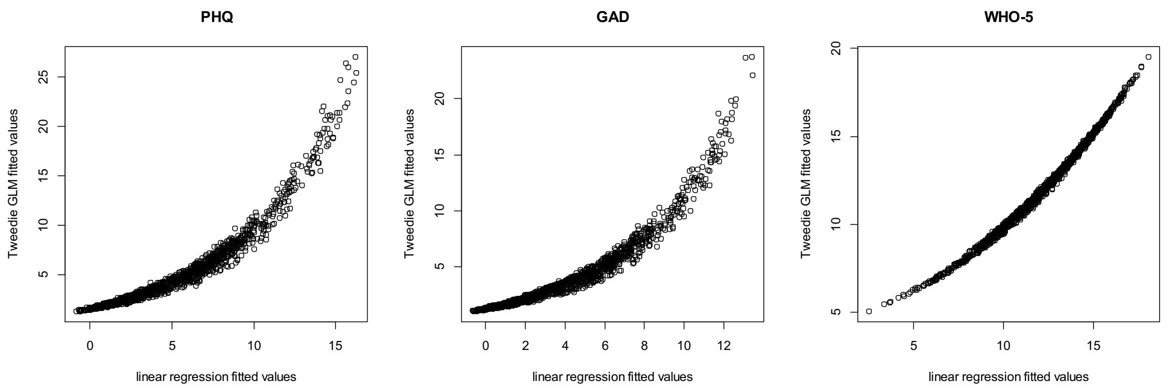

Imputed Data

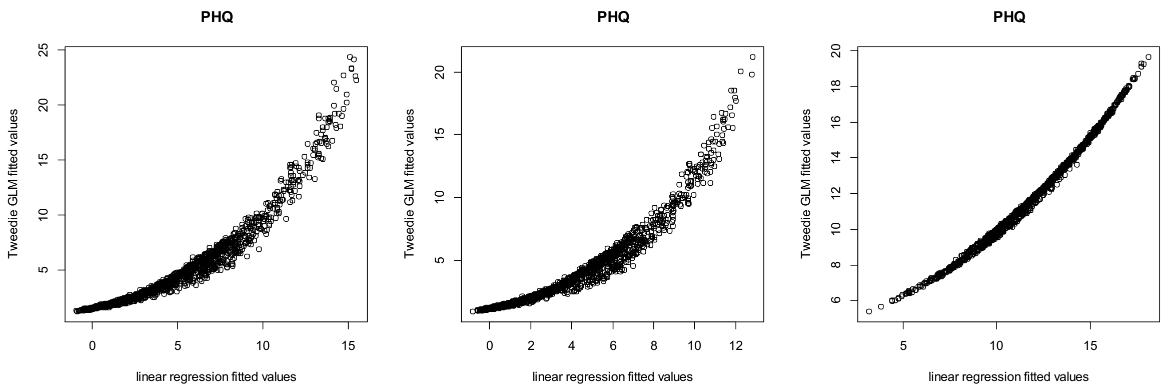

Figure S3b. Scatterplots of the Fitted Values for the Linear and Tweedie Models

Supplement S4 — Cross-Validation

Three of the independent variables in the multivariate regression models had relatively low-frequency categories, thereby potentially resulting in unstable models. These variables were whether the respondent lost their job due to COVID-19 ($N = 117$), whether they had direct exposure to COVID-19 ($N = 111$), and whether they had bushfire experience directly via fire ($N = 36$). We ran leave-one-out cross-validations for these three variables. That is, the regression models were run with each relevant case removed from the data, and that variable's coefficients, standard errors, and significance-levels were checked for evidence of instability. Table S4a displays the coefficients and significance-level ranges and quartiles for all of the cross-validation runs. There were no indications of instability or consequential variation in these results.

Table S4a. Cross-Validation Regression Coefficient and *p*-Value Ranges and Quartiles

		min	25%	50%	75%	max
**Job loss**						
**PHQ**	coeff.	.091	.185	.221	.252	.319
	p-value	.513	.606	.650	.705	.852
**GAD**	coeff.	.306	.391	.433	.456	.510
	p-value	.247	.303	.327	.376	.487
**WHO**	coeff.	-.485	-.410	-.364	-.333	-.268
	p-value	.382	.461	.515	.550	.630
**COVID-19 exposure**						
**PHQ**	coeff.	.188	.211	.215	.220	.318
	p-value	.513	.651	.659	.665	.699
**GAD**	coeff.	.385	.417	.420	.424	.510
	p-value	.247	.336	.340	.344	.384
**WHO**	coeff.	-.470	-.372	-.369	-.356	-.318
	p-value	.398	.502	.507	.522	.568
**Bushfire**						
**PHQ**	coeff.	.132	.211	.215	.219	.318
	p-value	.513	.653	.659	.664	.787
**GAD**	coeff.	.341	.419	.421	.424	.510
	p-value	.247	.336	.340	.342	.440
**WHO**	coeff.	-.485	-.374	-.371	-.366	-.285
	p-value	.382	.500	.504	.510	.609

Supplement S5 — Multiple Imputation Method and Models

Missing values (<1% of all variables analysed) were multiply imputed (10 iterations) by chained equations in R (version 3.6.3) with the package "mice" using the "pmm", "logreg", "polyreg", and "polr" algorithms for continuous, dichotomous, unordered categorical and ordered categorical variables respectively. In addition to non-imputed analyses, additional analyses were run on the multiply imputed dataset and pooled. Summary sample characteristics based on imputed data were computed on the aggregated multiply imputed datasets, as shown in Table S5a.

Table S5a Description of Sample Characteristics for Imputed Dataset

	Whole sample (n=1296)	Men (n=645)	Women (n=649)	*t* or χ2	*p*
**Sociodemographic and background factors**					
Age, years (SD)	46.04 (17.26)	49.45 (18.16)	42.67 (15.62)	7.19	**<.001*** **
Education, years (SD)	13.75 (2.59)	13.63 (2.65)	13.87 (2.52)	-1.70	.089
Has partner, n (%)	853 (66.23%)	421 (65.68%)	432 (66.77%)	.13	.723
Lives alone, n (%)	157 (12.11%)	82 (12.71%)	75 (11.52%)	.33	.567
Child at home, n (%)	406 (31.33%)	196 (3.39%)	210 (32.26%)	.44	.505
Any chronic disease, n (%)	503 (38.81%)	286 (44.34%)	217 (33.33%)	16.07	**<.001*** **
Any neurological disorder, n (%)	159 (12.27%)	86 (13.33%)	73 (11.21%)	1.16	.281
Any current MH disorder, n (%)	310 (23.92%)	144 (22.33%)	166 (25.50%)	1.62	.203
**Recent adversity**					
Bushfire exposure—smoke, n (%)	607 (46.84%)	290 (44.96%)	317 (48.69%)	1.67	.197
Bushfire exposure—fire, n (%)	111 (8.56%)	66 (1.23%)	45 (6.91%)	4.15	**.042***
Other adverse life event n (%)	282 (21.76%)	156 (24.19%)	126 (19.35%)	4.16	**.041***
**COVID-19 exposure**					
COVID-19 exposure, n (SD)	.78 (.88)	.71 (.82)	.85 (.93)	-2.73	**.006****
**Work and social impacts of COVID-19**					
Working from home, n (%)	173 (13.35%)	78 (12.09%)	95 (14.59%)	1.54	.214
Lost job, n (%)	117 (9.03%)	50 (7.75%)	67 (1.29%)	2.25	.134
Financial distress, n (%)	652 (5.31%)	314 (48.68%)	338 (51.92%)	1.23	.267
WSAS, n (SD)	2.54 (9.28)	2.25 (9.78)	2.82 (8.75)	-1.10	.271
**Mental health measures**					
PHQ9, score (SD)	5.37 (5.92)	4.73 (5.73)	6.01 (6.03)	-3.93	**<.001*** **
GAD7, score (SD)	4.40 (5.18)	3.66 (4.84)	5.13 (5.41)	-5.15	**<.001*** **
WHO5, score (SD)	11.90 (5.94)	12.90 (6.00)	10.91 (5.71)	6.11	**<.001*** **

Notes. *p<.05. **p<.001. ***p<.001.

Table S5b displays the linear regression coefficients and related statistics for the linear regression model using the imputed data. These are similar to and consistent with the results of the linear regression model in the main part of the paper, which used the non-imputed data.

Table S5b Linear Regression Models for Each Mental Health Outcome, for Imputed Dataset (for all models, *n*=1296, *df*=16, 1279)

	PHQ-9 estimate	*p*	GAD-7 estimate	*p*	WHO-5 estimate	*p*
**Constant**	3.57	**<.001*****	2.18	.018	12.38	**<.001*****
**Sociodemographic and background factors**						
Age	-.05	**<.001*****	-.04	**<.001*****	.03	**.003****
Gender	.85	**.002****	1.05	**<.001*****	-1.71	**<.001*****
Education	-.10	.053	-.04	.363	.15	**.015***
Has partner	-.39	.227	.20	.491	.61	.090
Lives alone	.35	.444	-.07	.876	-.24	.641
Child at home	-.28	.362	-.04	.897	.51	.137
Any chronic disease	.61	.060	.52	.080	-.81	.029
Any neurological disorder	1.33	**.004****	.45	.280	-.53	.300
Any current MH disorder	4.64	**<.001*****	3.94	**<.001*****	-3.06	**<.001*****
**Recent adversity**						
Bushfire exposure—smoke	.23	.378	.11	.662	-.95	**.002****
Bushfire exposure—fire	-.33	.498	-.51	.239	.67	.213
Other adverse life event	1.81	**<.001*****	1.33	**<.001*****	-.25	.516
**COVID-19 exposure**						
COVID-19 exposure	.24	.118	.18	.205	.39	.025
**Work and social impacts of COVID-19**						
Lost job	.37	.440	.47	.281	-.24	.658
Financial distress	2.32	**<.001*****	2.08	**<.001*****	-2.36	**<.001*****
WSAS	.09	**<.001*****	.06	**<.001*****	-.06	**.002****

	R^2	Adjusted R^2	F	R^2	Adjusted R^2	F	R^2	Adjusted R^2	F
**Model**	.371	.363	**47.19*****	.325	.317	**37.25*****	.207	.197	**20.86*****

Notes. **p*<.017. ***p*<.001. ****p*<.001.

Supplement S6 — Reliability analysis and results of univariate analysis

Table S6a contains several measures of internal consistency for the mental health and wellbeing scales employed as dependent variables in this paper. The first two columns report Cronbach's alpha and McDonald's omega, both of which are quite strong. The remaining columns report fit measures from a one-factor CFA, all of which indicate that a single-factor model adequately fits each of the scales.

Table S6a Internal Consistency Measures for the PHQ, GAD, and WHO Scales

	Alpha	Omega	vaccount	CFI	TLI	RMSEA
**PHQ**	.917	.917	.553	.982	.976	.097
**GAD**	.940	.940	.692	.995	.993	.095
**WHO**	.913	.914	.680	.964	.929	.106

Table S6b displays the univariate regression effects for models estimated on the non-imputed data.

Table S6b Univariate Analysis for Each Mental Health Outcome, Using Non-Imputed Dataset

	PHQ-9				GAD-7				WHO-5			
	estimate	*SE*	*statistic*	*p*	*estimate*	*SE*	*statistic*	*p*	*estimate*	*SE*	*statistic*	*p*
Age	-.09	.01	-1.31	**<.001******	-.08	.01	-1.52	**<.001******	.07	.01	6.97	**<.001******
Gender (ref=female)	1.29	.33	3.93	**<.001******	1.45	.29	5.07	**<.001******	-2.01	.33	-6.16	**<.001******
Education, years	-.08	.06	-1.24	.215	-.01	.06	-.12	.907	.14	.06	2.15	.032
Has partner	-1.05	.35	-3.02	**.003****	-.17	.31	-.57	.570	1.06	.35	3.03	**.003****
Lives alone	-.17	.51	-.35	.729	-.96	.44	-2.16	.031	-.23	.51	-.46	.649
Child at home	.52	.35	1.45	.147	.85	.31	2.76	**.006****	.02	.36	.06	.954
Any chronic disease	.86	.34	2.56	**.011***	.41	.30	1.39	.165	-.71	.34	-2.10	.036
Any neurological disorder	3.38	.49	6.85	**<.001******	2.00	.44	4.59	**<.001******	-1.99	.50	-3.98	**<.001******
Any current MH disorder	5.93	.35	17.01	**<.001******	4.79	.31	15.41	**<.001******	-3.95	.37	-1.62	**<.001******
Bushfire exposure—smoke	.85	.33	2.59	**.010***	.61	.29	2.12	.034	-1.26	.33	-3.81	**<.001******
Bushfire exposure—fire	1.65	.59	2.81	**.005****	1.07	.52	2.07	.039	-.40	.59	-.67	.502
Other adverse life event	4.00	.38	10.45	**<.001******	3.06	.34	9.00	**<.001******	-1.74	.40	-4.38	**<.001******
COVID-19 exposure	.91	.19	4.90	**<.001******	.74	.16	4.56	**<.001******	-.10	.19	-.51	.611
Working from home	-.53	.48	-1.09	.273	.18	.42	.43	.665	.34	.49	.71	.478
Lost job	2.96	.57	5.21	**<.001******	2.65	.50	5.32	**<.001******	-2.10	.57	-3.66	**<.001******
Financial distress	4.24	.31	13.77	**<.001******	3.64	.27	13.48	**<.001******	-3.34	.32	-10.53	**<.001******
WSAS	.22	.02	13.01	**<.001******	.18	.01	11.91	**<.001******	-.14	.02	-8.18	**<.001******

Notes. *p<.017. **p<.001. ***p<.001.

References

R Core Team (2013) *R: A language and environment for statistical computing.* Vienna: R Foundation for Statistical Computing.

M. Smithson, and Y. Shou (2019) *Generalized linear models for bounded and limited quantitative variables.* SAGE Publications.

S. van Buuren, and K. Groothuis-Oudshoorn (2009) *MICE: Multivariate Imputation by Chained Equations in R Journal of Statistical Software, forthcoming*. Available at: http://CRAN. R-project. org/package=mice.

Y. Zhang (2013) Likelihood-based and bayesian methods for tweedie compound poisson linear mixed models. *Statistics and Computing* 23(6): 743–757.
